# Development, environmental degradation, and disease spread in the Brazilian Amazon

**DOI:** 10.1371/journal.pbio.3000526

**Published:** 2019-11-15

**Authors:** Marcia C. Castro, Andres Baeza, Cláudia Torres Codeço, Zulma M. Cucunubá, Ana Paula Dal’Asta, Giulio A. De Leo, Andrew P. Dobson, Gabriel Carrasco-Escobar, Raquel Martins Lana, Rachel Lowe, Antonio Miguel Vieira Monteiro, Mercedes Pascual, Mauricio Santos-Vega

**Affiliations:** 1 Department of Global Health and Population, Harvard T.H. Chan School of Public Health, Boston, Massachusetts, United States of America; 2 Center for Global Discovery and Conservation Science (GDCS), Arizona State University, Tempe, Arizona, United States of America; 3 Programa de Computação Científica, Fundação Oswaldo Cruz, Rio de Janeiro, Brazil; 4 MRC Centre for Global Infectious Disease Analysis (MRC GIDA), Department of Infectious Disease Epidemiology, Imperial College London, London, United Kingdom; 5 Instituto Nacional de Pesquisas Espaciais, São José dos Campos, São Paulo, Brazil; 6 Woods Institute for the Environment and Hopkins Marine Station of Stanford University, Pacific Grove, California, United States of America; 7 Department of Ecology and Evolutionary Biology, Princeton University, Princeton, New Jersey, United States of America; 8 Institute of Tropical Medicine “Alexander von Humboldt,” Universidad Peruana Cayetano Heredia, Lima, Peru; 9 Centre on Climate Change and Planetary Health & Centre for Mathematical Modelling of Infectious Diseases, London School of Hygiene & Tropical Medicine, London, United Kingdom; 10 Barcelona Institute for Global Health, Barcelona, Spain; 11 Department of Ecology and Evolution, University of Chicago, Chicago, Illinois, United States of America; 12 Departamento de Ingeniería Biomédica, Grupo de Investigación en Biología Matemática y Computacional BIOMAC, Universidad de los Andes, Bogotá, Colombia

## Abstract

The Amazon is Brazil’s greatest natural resource and invaluable to the rest of the world as a buffer against climate change. The recent election of Brazil’s president brought disputes over development plans for the region back into the spotlight. Historically, the development model for the Amazon has focused on exploitation of natural resources, resulting in environmental degradation, particularly deforestation. Although considerable attention has focused on the long-term global cost of “losing the Amazon,” too little attention has focused on the emergence and reemergence of vector-borne diseases that directly impact the local population, with spillover effects to other neighboring areas. We discuss the impact of Amazon development models on human health, with a focus on vector-borne disease risk. We outline policy actions that could mitigate these negative impacts while creating opportunities for environmentally sensitive economic activities.

## Abstract 2

This Perspective article maintains that a development model for the Amazon must be bold, creative, carefully planned, and sustainable, explicitly acknowledging the importance of public health in reinforcing economic development, environmental protection, and land use change.

## Development, environmental degradation, and disease spread in the Brazilian Amazon

The Amazon basin is changing rapidly. At present, it covers 7% of the Earth’s surface and 40% of the South American continent. It is home to approximately 38 million people living in one of the most biodiverse regions on Earth. Although the Amazon is shared between nine countries (Bolivia, Brazil, Colombia, Ecuador, French Guiana, Guyana, Peru, Suriname, and Venezuela) almost 70% resides in Brazil. It holds the largest megadiverse tropical rainforest in the world, a vast amount of natural resources (e.g., hydropower, minerals, timber), and a predominantly unexplored source of new bioactive compounds for the pharmaceutical, cosmetic, and botanical industry. It also plays a crucial role in regulating local and regional weather patterns and acts as a buffer against global climate change [1]. This rich natural environment contrasts with the living conditions of the Brazilian Amazonians: the burden of infectious diseases is very high (e.g., the region concentrates about 99% of malaria cases in the country), and life expectancy is 5 years lower than in the more developed Southeast region.

**Fig 1:**
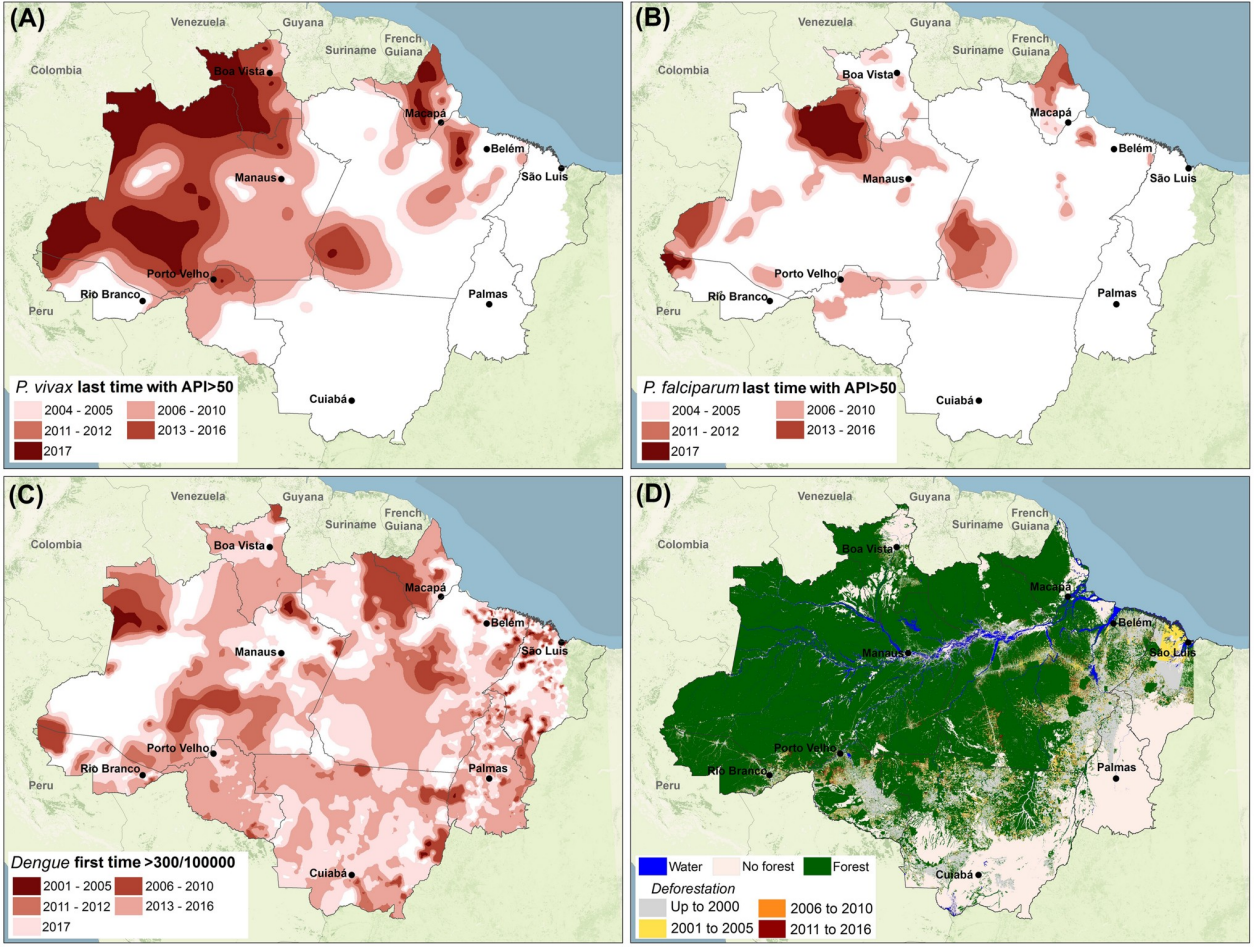
Changes in infectious disease transmission intensity and deforestation in the Brazilian Amazon. Hotspots of malaria transmission for **(A)** *Plasmodium vivax* and **(B)** *P*. *falciparum* in the Brazilian Amazon. The color intervals correspond to the spatial mean (ordinary kriging) of the last time period with API greater than 50 between 2004 and 2017. Monthly malaria notification data at the municipality level were obtained from SIVEP/Malaria. **(C)** Expansion of dengue transmission across the Brazilian Amazon. The color intervals correspond to the spatial mean (ordinary kriging) of the first time period with a high incidence rate (more than 300 cases per 100,000 inhabitants) between 2001 and 2017 [6]. Monthly dengue notification data at the municipality level were obtained from SINAN. **(D)** Deforestation over three time periods between 2001 and 2016. The category “No forest” refers to areas that are part of the Amazon biome but are covered by distinct types of vegetation mainly savanna. Data are available at http://www.obt.inpe.br/prodes/. *This figure was created in ArcGIS version (ESRI; Redlands*, *CA*, *USA)*. *Base map*: *MODIS-derived product*, *MOD13A2 EVI (USGS/NASA); administrative boundaries available from the Brazilian Institute of Geography and Statistics (IBGE*, *2010 –* https://mapas.ibge.gov.br/bases-e-referenciais/bases-cartograficas/malhas-digitais*)*. API, Annual Parasite Index; SINAN, Notifiable Diseases Information System; SIVEP/Malaria, Brazilian Epidemiological Surveillance Information System for Malaria.

Development models for the Brazilian Amazon varied from top-down initiatives, such as large industrial, agrobusinesses, and infrastructure projects, to bottom-up initiatives based on the organization of local production chains and market establishment based on forest products [2]. Economic and population booms, driven mainly by cycles of resource exploitation, promoted the Brazilian Amazon into an area of geopolitical strategic importance in the mid-1960s. Development policies strived for regional integration, national security, and resource exploitation. This was accompanied by the opening of highways, construction of dams, tax subsidies for the agroindustry, and promotion of agricultural settlements that brought formidable landscape changes to the region. Economic migration brought millions of people in search of land, and population growth in the Brazilian Amazon increased sharply (the proportion of the Brazilian population living in the Amazon increased from 7.4% in 1950 to 13.3% in 2010). Part of this growth resulted in new cities: the 2010 Population Census showed that 10 of the 19 cities that doubled in size between 2000 and 2010 were in the Amazon, and urbanization reached 71.8%, providing evidence of a move toward an “urbanized forest” [3]. However, these changes fell short in promoting sustainable development, and the region witnessed a dramatic increase in deforestation rates (approximately 20% of the forest cover has been removed—Fig 1D) and in the incidence of infectious diseases [4, 5].

Brazil’s new government has renewed concerns of unsustainable economic development [7]. Different forest monitoring and alert systems have pointed consistently to a significantly average rise in the deforestation rate in 2019 [8, 9]. Large-scale projects are already intensifying resource extraction and land conversion, including, for example, the Barão do Rio Branco Project, which encompasses the construction of a large hydropower plant in the Trombetas River, a bridge over the Amazon River, a road from Belém to Suriname, and the opening of previously protected areas (including indigenous reserves) for intensive mining of copper and rare metals, such as niobium. These actions (planned and ongoing) have consequences that extend beyond Brazil and the Amazon borders [7]. Political decisions in Brazil are likely to resonate across the region. For example, the impacts of altered climate regimes will be felt regionally, and changes in infectious disease risk also threaten neighboring countries.

Forest degradation in the Amazon has facilitated the spread of diseases with potentially large social and economic impacts, both locally and globally. Multiple pathogens thrive under land-use changes, deforestation, and poverty, causing a significant burden to the health and economic prosperity of Amazonians. The health dimension is scarcely included in discussions around development of the region. Here, we briefly discuss how historical colonization processes have had a negative impact on vector-borne disease emergence and reemergence in Brazil. We then outline policy actions that can concomitantly mitigate the negative impacts of the Amazon’s development on biodiversity and the global climate system while creating opportunities for economic productivity. These policies reduce the detrimental health impacts for the expanding population of the Amazon.

### Trends in disease burden

Environmental changes observed in the Brazilian Amazon since the late 1970s were accompanied by a new disease burden profile. The opening of new agricultural settlements intensified during the 1980s, and these were invariably accompanied by malaria outbreaks [10]. Entomologically, the process of forest clearing created ideal conditions for the malaria vector (*Anopheles* mosquito): clean, partly sunlit, and clear water with pH near neutral (as opposed to acidic water found in the undisturbed forest) [5]. The context and dynamics of transmission in those settings were unique and defined as “frontier malaria” [11, 12] to characterize the importance of biological, ecological, and sociodemographic factors operating over time at three spatial scales: micro/individual, community, and state/national. Here, the temporal component was critical, as a transition was often observed from an initial epidemic phase to endemic malaria transmission, with a transient but long-lasting increase in total burden that extended over a period of about 8–10 years after the opening of a new settlement [13]. This transition reflected changes in socioeconomic conditions but also in environmental modifications (a period of intense deforestation a few years after occupation, followed by much lower changes in forest cover) [5].

Indeed, the number of malaria cases in the Brazilian Amazon increased sharply after the late 1970s, with a peak of 632,800 cases in 1999. This decadal temporal pattern plays out in space tracking the expansion of deforestation (Fig 1). The outbreak of malaria following deforestation in frontier regions is also the most common trajectory predicted by theory based on mathematical models including bidirectional feedbacks between malaria incidence, capital accumulation, and land-use change [14]. In addition to deforestation itself, some attempts to improve the livelihoods of the Amazonians had further unanticipated health consequences. In the early 2000s, fish farming activities were promoted and subsidized to improve livelihoods and stimulate the local economy. The physical characteristics of fish ponds were conducive to *Anopheles* breeding, resulting in an increase in malaria cases [15]. Different control programs were implemented to curb the increase in malaria cases of the 1990s and 2000s [16–18]. These promoted varied actions (e.g., improved network of testing laboratories, rapid diagnosis and treatment, focal use of bednets, capacity building) and resulted in a steady decline in transmission from 2005 to 2016, reaching a minimum of 130,000 cases in 2016—the lowest number recorded in 38 years. Importantly, however, this trend has recently reversed, with transmission increasing by 50% between 2016 and 2018, which undermines *P*. *falciparum* elimination goals set in 2015 [17].

In addition, unplanned and precarious urbanization that lacks basic infrastructure (e.g., regular access to water and waste collection), associated with change of habits and customs of the Amazonian population, created ideal conditions for *Aedes aegypti*, the main vector of dengue, chikungunya, and Zika viruses. This facilitated the rapid spread of these diseases across the Brazilian Amazon, with an approximate doubling of the percent of municipalities reporting dengue in 15 years (40% to 85% from 2000 to 2014). Their coexistence and nonspecific clinical manifestations pose unprecedented challenges for accurate diagnoses and treatment (Fig 1C). City expansion has also favored the urbanization and reemergence of Chagas disease [19], mainly through oral transmission, which has been linked to the consumption of local açai berries [19]. Currently, 95% of Brazilian cases of Chagas occur in the Amazon, and about 73% occur via oral transmission.

Actions to address deforestation, the loss of biodiversity, and to protect human health included (1) the introduction of forest and infectious disease monitoring systems, (2) the requirement of an environmental impact assessment (EIA) for major infrastructure projects, (3) demarcation of indigenous areas and forest reserves, and (4) the establishment of research institutions in the area [20]. Yet many factors contribute to a disconnect between planned and real actions, such as a lack of accountability, transparency, and little consideration of health in EIAs [20]. There is increasing scientific consensus that continued deforestation in the region facilitates an increase in disease risk from vector-borne pathogens [21]; furthermore, its interaction with the climate can create significantly prolonged droughts, reducing productivity and minimizing the viability of agriculture in the region [22].

### Policy implications

The feedback between development policies and disease burden affects local productivity, human capital, and livelihoods [23]. Three strategies could help promote improved policies for the Brazilian Amazon.

**First, it is imperative to maintain, reorganize, and integrate existing efforts in monitoring land-use change and in surveillance and control of infectious diseases**. For example, the Instituto Nacional de Pesquisas Espaciais (INPE)’s Detection of Deforestation in Real Time (DETER) identifies areas in the Brazilian Amazon where changes in forest cover have occurred (http://www.obt.inpe.br/deter/). MapBiomas (http://alerta.mapbiomas.org/en) is an example of a successful partnership between Brazilian and international institutions, built upon DETER to integrate environmental, infrastructure, and administrative data into a single platform, which has already released 33 years (for the years 1985 to 2017) of open-access data on annual land cover and land-use changes. DETER and MapBiomas should be integrated with administrative records systematically collected by the Ministry of Health. Incorporating epidemiological alerts in these systems could allow a shift from reactive to proactive disease management. Given the link between environmental change and malaria [21], DETER/MapBiomas could serve as an additional surveillance tool for the National Malaria Control Program (NMCP), aimed at curbing local outbreaks. Most importantly, this strategy does not require new financial resources as DETER/MapBiomas are fully developed systems, the communication of reports to the NMCP is an easy process, and action on those reports locally would not involve new activities but instead involve an optimization of existing local ones (from reactive to proactive). Along the same lines, ongoing efforts in Brazil to provide real-time surveillance of arboviruses using meteorological, epidemiological, and social media data (info.dengue.mat.br) could be easily expanded to the Amazon region.

In addition, the Brazilian Agricultural Research Corporation (EMBRAPA) has developed protocols to treat açai berries before processing in order to avoid contamination by Chagas disease vectors. Dissemination of this knowledge to local producers and promotion of local cooperatives to minimize the cost of treating the berries are of utmost importance and would be viable through a partnership between agriculture, health, and commerce sectors, leveraging existing local committees that bring together different stakeholders (e.g., National Council of Municipal Health Secretariats [24]). More broadly, the integration of environmental, health, and socioeconomic data must be the basis for developing local surveillance tools and alert systems to increase the effectiveness of disease control strategies [25]. Finally, including information from other countries would greatly increase the capacity to monitor the Amazon basin as a system and respond to cross-border threats and challenges.

**Second, planned action in the Amazon basin should consider the regional perspective**. Instability in neighboring countries may jeopardize actions taken to achieve sustainable development. For example, the ongoing crisis in Venezuela has resulted in environmental degradation due to illegal mining and the exportation of measles and malaria cases to other countries in the South America [26]. Coordinated efforts for conservation, improvement of social well-being, and implementation of standardized protocols for disease diagnosis, treatment, and control should be rigorously pursued. A regional agreement to regulate native vegetation protection and land use in the Amazon basin should be developed [27]. Also, strengthening of the Proyecto de Monitoreo de la Cobertura Forestal en la Región Amazónica (Forest Monitoring over the Amazonian Region Project), which was implemented in 2011 by the Organización del Tratado de Cooperación Amazónica (OTCA, Amazon Cooperation Treaty Organization) in partnership with INPE would help build a common regional base for a forest monitoring system. Similarly, the Amazon Malaria Initiative (an 11-country regional program in the Amazon basin and Central America) and the Amazon Network for the Surveillance of Antimalarial Drug Resistance (a network organized in 2001 by Bolivia, Brazil, Colombia, Ecuador, Guyana, Peru, Suriname, and Venezuela, along with the Pan American Health Organization (PAHO), in response to the challenge of antimalarial drug resistance in the Amazon) are examples of the feasibility of regional actions. New and expanded regional collaborations should be pursued by disease control programs and institutions, facilitated and supported by intergovernmental organizations (e.g., PAHO).

**Third, revisiting austerity measures that affect health and the environment, in light of international evidence regarding their negative consequences, is needed to avoid irreversible destruction of the Amazon**. In 2016, the Brazilian government imposed a strict limit to the growth of public expenditure over the next 20 years to a level based on the value of its previous financial year adjusted for inflation, threatening the sustainability of the health system [28]. Concomitantly, there is pressure from the government to change environmental legislation, which could weaken conservation efforts [29]. These factors could reverse decades of improvement in health outcomes and deforestation reduction, potentially leading to a devastating scenario of environmental loss and increased inequalities, as observed in Brazil in the 1980s and early 1990s (when austerity measures were also implemented) [30, 31]. Solid scientific evidence of the possible consequences, engagement of different actors, and population pressure on constituents are necessary but not sufficient to promote change, given the prevailing neoliberal ideals that support and promote austerity measures, despite widespread and increasing inequality [31, 32]. Although an economic recession is often accompanied by unpopular and controversial decisions, measures implemented so far address neither indirect and regressive taxation [33] nor distorted salary and benefits to some government branches [34]. Austerity measures do not solve these fundamental distortions. Measures to mitigate them would translate into better allocation of financial resources and, in doing so, contribute to a reduction in inequalities and make cuts in health expenditure avoidable.

The Amazon’s vibrant and rich species diversity masks the underlying fragility of the poor soils and the vulnerability of its people to environmental changes and infectious diseases. A development model for the Amazon must learn from history and be bold, creative, carefully planned, inclusive, and sustainable, allowing for distributive economic growth while avoiding environmental, social, and health problems. It can also provide a vital template for other nations that look to Brazil for leadership on how development can proceed in ways that benefit local people and national economies. Indeed, Brazil played a major role in setting the agenda for sustainable development when it hosted the Biodiversity Convention in 1992. Twenty-seven years later, the country is under the leadership of a new government that favors the opening of protected areas to agriculture and mining—which has been referred to as a “death agenda” [7], based on activities historically associated with deforestation and increases in disease burden (e.g., malaria) [35]. Despite these challenging political circumstances, pursuing our policy strategies should commence through the initiative of specific governmental sectors. The current political landscape demands active engagement of civil society and academia (both from Brazil and abroad) with constituents in the Congress and Senate to protect the Amazon rainforest and to provide viable solutions for sustainable and responsible economic growth, prosperity, and well-being in the region.

## Abbreviations

DETER: Detection of Deforestation in Real Time
EIA: environmental impact assessment
EMBRAPA: Brazilian Agricultural Research Corporation
INPE: Instituto Nacional de Pesquisas Espaciais
NMCP: National Malaria Control Program
OTCA: Organización del Tratado de Cooperación Amazónica
PAHO: Pan American Health Organization

## References

[1] Nobre CA, Sampaio G, Borma LS, Castilla-Rubio JC, Silva JS, Cardoso M. Land-use and climate change risks in the Amazon and the need of a novel sustainable development paradigm. Proceedings of the National Academy of Sciences. 2016;113(39):10759–68. 10.1073/pnas.1605516113 27638214 PMC5047175

[2] Porto L, Macedo FC, editors. Existe uma Política Nacional de Desenvolvimento Regional no Brasil? Territórios, Redes e Desenvolvimento Regional: Perspectivas e Desafios; 2017 Sep 13–15; Santa Cruz do Sul, RS, Brasil.

[3] Becker BK. Undoing Myths: the Amazon—an urbanized forest In: Clusener-Godt M, Sachs I, editors. Brazilian Perspectives on Sustainable Development of the Amazon Region. Paris, France: UNESCO and The Parthenon Publishing Group; 1995 p. 53–89.

[4] Hahn MB, Gangnon RE, Barcellos C, Asner GP, Patz JA. Influence of Deforestation, Logging, and Fire on Malaria in the Brazilian Amazon. PLoS ONE. 2014;9(1):e85725 10.1371/journal.pone.0085725 24404206 PMC3880339

[5] Sawyer DR. Malaria and the Environment. Documento de Trabalho. Brasília: Instituto SPN; 1992 Mar.

[6] Barcellos C, Lowe R. Expansion of the dengue transmission area in Brazil: the role of climate and cities. Tropical Medicine & International Health. 2014;19(2):159–68. 10.1111/tmi.12227 24286460

[7] Ferrante L, Fearnside PM. Brazil’s new president and ‘ruralists’ threaten Amazonia’s environment, traditional peoples and the global climate. Environmental Conservation. 2019; 46(4):261–263. 10.1017/S0376892919000213

[8] Fonseca A, Justino M, Cardoso D, Ribeiro J, Salomão R, Jr. S, et al. Boletim do desmatamento da Amazônia Legal (julho 2019) SAD. Belém: Imazon. [cited 2019 August 12]. https://imazon.org.br/publicacoes/boletim-do-desmatamento-da-amazonia-legal-julho-2019-sad/

[9] INPE. DETER, 2019. Instituto Nacional de Pesquisas Espaciais, 2019. [cited 2019 August 12]. http://terrabrasilis.dpi.inpe.br/

[10] Sawyer DR. Malaria and the Environment. Brasília: Instituto SPN; 1992.

[11] Castro MC, Monte-Mór RL, Sawyer DO, Singer BH. Malaria Risk on the Amazon Frontier. Proceedings of the National Academy of Sciences. 2006;103(7):2452–7. 10.1073/pnas.0510576103 PMC1413719 16461902

[12] Sawyer DR. Malaria on the Amazon frontier: economic and social aspects of transmission and control. Southeast Asian Journal of Tropical Medicine and Public Health. 1986;17(3):342–5. 3563600

[13] Sawyer DR, Sawyer DO. The malaria transition and the role of social science research In: Chen LC, editor. Advancing the health in developing countries: the role of social research. Westport: Auburn House; 1992 p. 105–22.

[14] Baeza A, Santos-Vega M, Dobson AP, Pascual M. The rise and fall of malaria under land-use change in frontier regions. Nature Ecology & Evolution. 2017;1:0108 Available from: https://www.nature.com/articles/s41559-017-0108#supplementary-information. 28812707 10.1038/s41559-017-0108

[15] Reis IC, Honório NA, Barros FSM, Barcellos C, Kitron U, Camara DCP, et al Epidemic and Endemic Malaria Transmission Related to Fish Farming Ponds in the Amazon Frontier. PLoS ONE. 2015;10(9):e0137521 10.1371/journal.pone.0137521 26361330 PMC4567347

[16] Carlos BC, Rona LDP, Christophides GK, Souza-Neto JA. A comprehensive analysis of malaria transmission in Brazil. Pathogens and Global Health. 2019;113(1):1–13. 10.1080/20477724.2019.1581463 30829565 PMC6425916

[17] Ferreira MU, Castro MC. Challenges for malaria elimination in Brazil. Malaria Journal. 2016;15:284 10.1186/s12936-016-1335-1 27206924 PMC4875681

[18] Griffing SM, Tauil PL, Udhayakumar V, Silva-Flannery L. A historical perspective on malaria control in Brazil. Mem Inst Oswaldo Cruz. 2015;110(6):701–18. 10.1590/0074-02760150041 . 26517649 PMC4667572

[19] Shikanai-Yasuda MA, Carvalho NB. Oral transmission of Chagas disease. Clinical infectious diseases: an official publication of the Infectious Diseases Society of America. 2012;54(6):845–52. Epub 2012/01/13. 10.1093/cid/cir956 . 22238161

[20] Fearnside PM. Environmental policy in Brazilian Amazonia: Lessons from recent history. Novos Cadernos NAEA. 2016;19(1):27–46. Available from: https://periodicos.ufpa.br/index.php/ncn/article/view/1379.

[21] Chaves LSM, Conn JE, López RVM, Sallum MAM. Abundance of impacted forest patches less than 5 km2 is a key driver of the incidence of malaria in Amazonian Brazil. Scientific Reports. 2018;8(1):7077 10.1038/s41598-018-25344-5 29728637 PMC5935754

[22] Malhi Y, Roberts JT, Betts RA, Killeen TJ, Li W, Nobre CA. Climate Change, Deforestation, and the Fate of the Amazon. Science. 2008;319(5860):169–72. 10.1126/science.1146961 18048654

[23] Rohr JR, Barrett CB, Civitello DJ, Craft ME, Delius B, DeLeo GA, et al Emerging human infectious diseases and the links to global food production. Nature Sustainability. 2019;2(6):445–56. 10.1038/s41893-019-0293-3 PMC7091874 32219187

[24] Martinez MG, Kohler JC. Civil society participation in the health system: the case of Brazil’s Health Councils. Globalization and Health. 2016;12(1):64 10.1186/s12992-016-0197-1 27782831 PMC5080747

[25] Coelho FC, Codeço CT. Precision epidemiology of arboviral diseases. Journal of Public Health and Emergency. 2019;3:1 10.21037/jphe.2018.12.03

[26] Grillet ME, Villegas L, Oletta JF, Tami A, Conn JE. Malaria in Venezuela requires response. Science. 2018;359(6375):528-. 10.1126/science.aar5440 29420282

[27] Kremen C, Merenlender AM. Landscapes that work for biodiversity and people. Science. 2018;362(6412):eaau6020. 10.1126/science.aau6020 30337381

[28] Castro MC, Massuda A, Almeida G, Menezes-Filho NA, Andrade MV, de Souza Noronha KVM, et al Brazil's unified health system: the first 30 years and prospects for the future. The Lancet. 2019;394(10195):345–56. 10.1016/S0140-6736(19)31243-7 31303318

[29] Tollefson J. Brazil's lawmakers renew push to weaken environmental rules. Nature. 2018;557(7703):17 Epub 2018/05/03. 10.1038/d41586-018-05022-2 . 29717253

[30] Santos IS, Vieira FS. The Right to healthcare and fiscal austerity: the Brazilian case from an international perspective. Ciência & Saúde Coletiva. 2018;23:2303–14. 30020383 10.1590/1413-81232018237.09192018

[31] Ostry JD, Loungani P, Furceri D. Neoliberalism: oversold? Finance & Development 2016;53(2):38–41.

[32] Stuckler D, Basu S. The Body Economic: Why Austerity Kills. New York, NY: Basic Books; 2013.

[33] Gobetti SW, Orair RO. Taxation and distribution of income in Brazil: new evidence from personal income tax data. Brazilian Journal of Political Economy. 2017;37(2):267–86.

[34] Dias M, Aylmer R. Is the Brazilian civil service reform about to succeed? Global Journal of Political Science and Administration. 2018;6(2):13–25.

[35] Fraser B. Taking on malaria in the Amazon. The Lancet. 2010;376(9747):1133–4. 10.1016/S0140-6736(10)61522-X 20922838

